# Supplementary material for: The Structure of Evolutionary Model Space for Proteins across the Tree of Life
Source: Biology (Basel). 2023 Feb 10;12(2):282. doi: 10.3390/biology12020282 (PMC9952988; doi:10.3390/biology12020282)
Supplement: Supplementary file 1 [file biology-12-00282-s001.zip › Scolaro_Braun_2022_supplementary_data/Scolaro_Braun_Supplementary_Figure_S2.pdf]

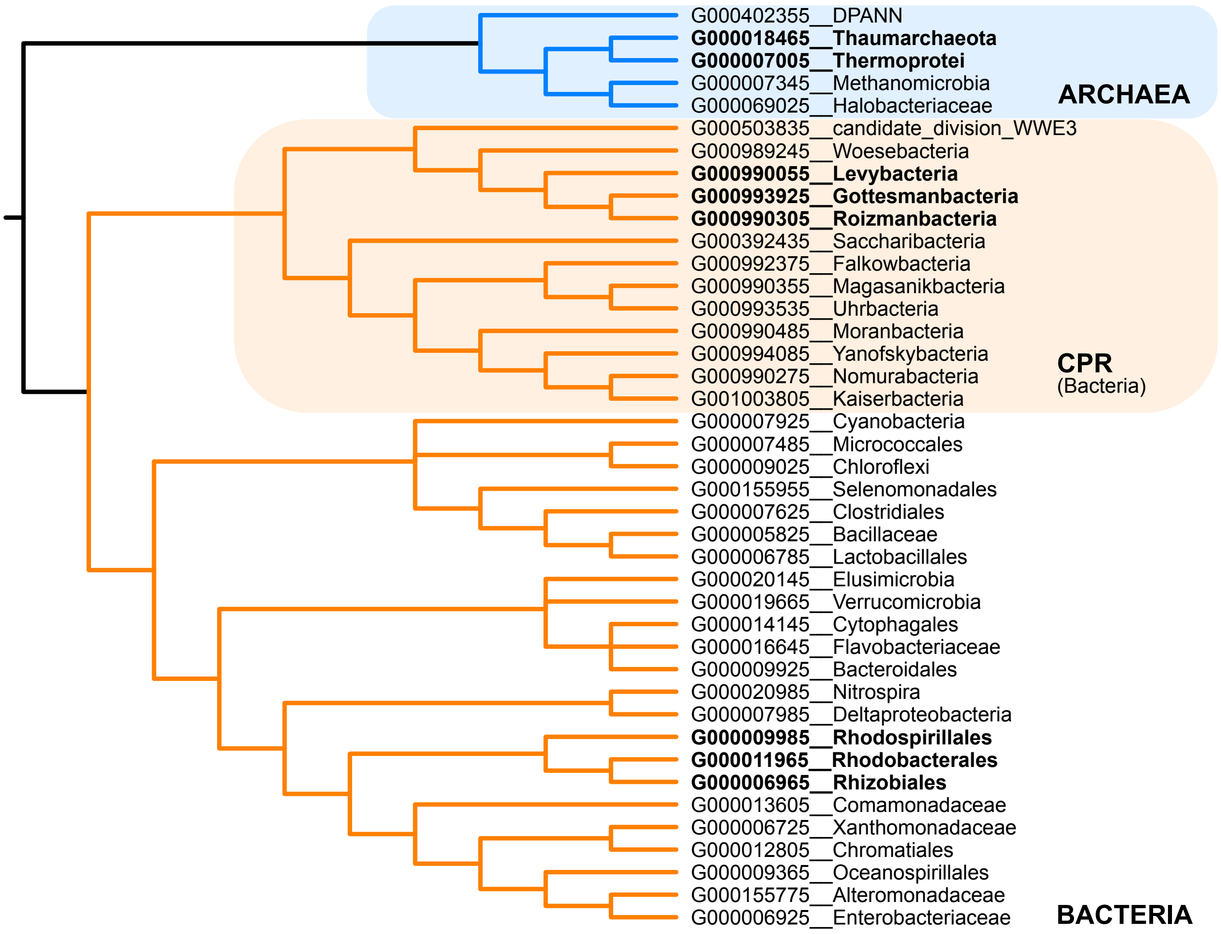

Supplementary Figure S2. Topology of the Zhu et al. [92] supertree, pruned to include the same prokaryotic taxa as the Hug tree shown in Figure 1c. Bacteria and Archaea are labeled using the same colors as Figure 1, although we have added orange highlighting for the bacterial CPR (candidate phyla radiation) in this tree. The bold taxa correspond to parts of the tree that have a topology identical to groups in the tree of models (see text); note that the Zhu et al. [92] supertree does not include eukaryotes, so they are not included in this figure. The "G" numbers are codes used in Zhu et al. [92] (see File02.tsv in the supplementary information for that paper). Taxon pruning was accomplished by modifying the taxon name for the first G number that belongs to the relevant group (i.e., the output of `grep "CLADENAME" File02.tsv | head -n +1`, using the clade names listed). Then, the appropriate G numbers for taxa in File01.nwk (from the Zhu et al. [92] supplementary information) were renamed, the newick tree with renamed taxa was converted to nexus format, and the other taxa in the tree were pruned using PAUP* 4.0a168 [41]. Reference number in this legend match the references listed in the main manuscript.
