# Supplementary material for: The Structure of Evolutionary Model Space for Proteins across the Tree of Life
Source: Biology (Basel). 2023 Feb 10;12(2):282. doi: 10.3390/biology12020282 (PMC9952988; doi:10.3390/biology12020282)
Supplement: Supplementary file 1 [file biology-12-00282-s001.zip › Scolaro_Braun_2022_supplementary_data/Scolaro_Braun_Supplementary_Figure_S1.pdf]

(a)

Estimated Equilibrium Frequencies of Aromatic Amino Acids

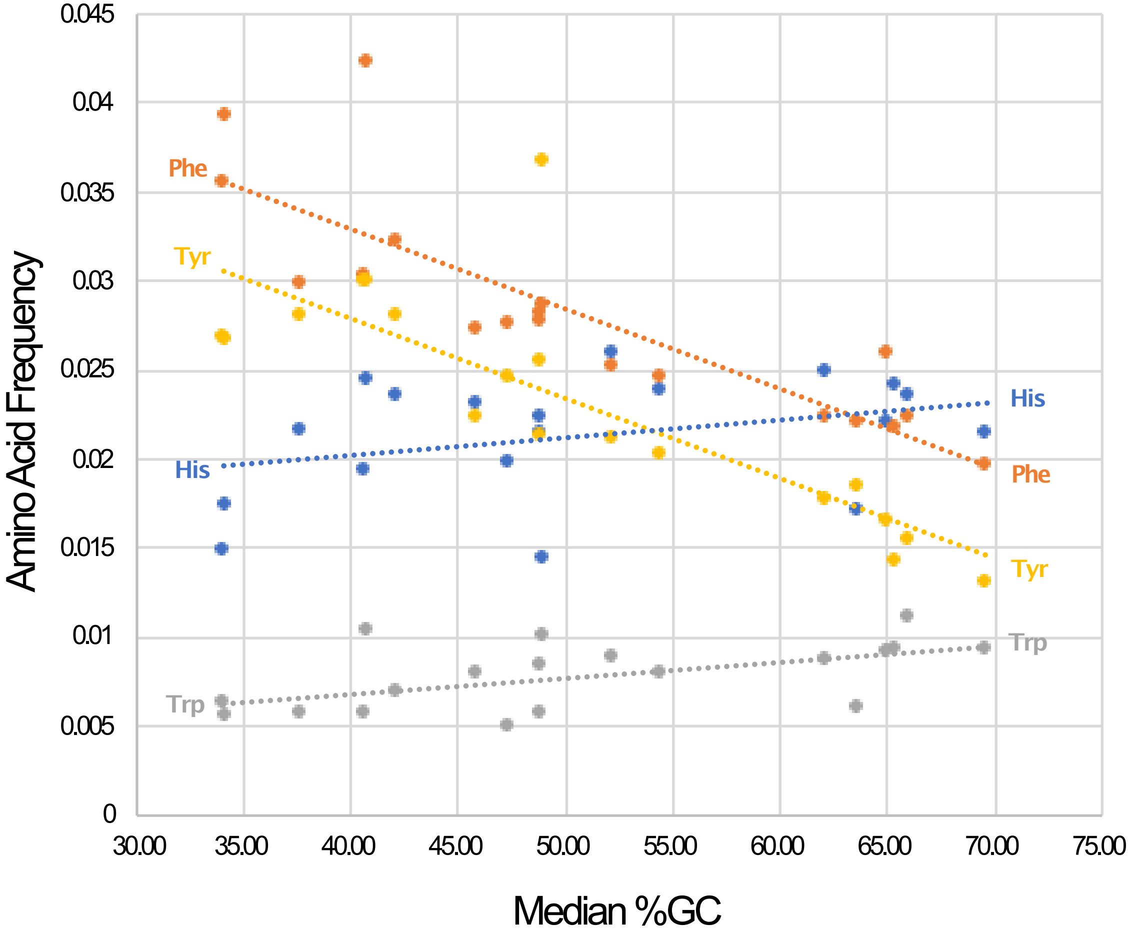

(b)

Correlations between Selected Amino Acid Frequency Estimates and Genomic GC-content

	Spearman's ρ	P (two-tailed)
His (H)	0.29574	0.21895
Phe (F)	-0.9118	<0.00001
Trp (W)	0.60728	0.00582
Tyr (Y)	-0.8416	0.00001
Ala (A)	0.9118	<0.00001
Cys (C)	-0.37209	0.11671
Ser (S)	-0.31856	0.18376
